# Supplementary material for: HIVprotI: an integrated web based platform for prediction and design of HIV proteins inhibitors
Source: J Cheminform. 2018 Mar 9;10:12. doi: 10.1186/s13321-018-0266-y (PMC5845081; doi:10.1186/s13321-018-0266-y)
Supplement: Supplementary file 2 — Additional file 2. Source code of HIVProtI web server. [file 13321_2018_266_MOESM2_ESM.zip › HIVprotI_Source-code/hivprotI/weka-3-6-6/doc/weka/core/Debug.Random.html]

Debug.Random


---


|  |  |  |
| --- | --- | --- |
| **Overview** \| **Package** \| **Class** \| **Tree** \| **Deprecated** \| **Index** \| **Help** \| **Weka's home** | |  |
| **PREV CLASS**   **NEXT CLASS** | **FRAMES**    **NO FRAMES**     **All Classes** |  |
| SUMMARY: NESTED \| FIELD \| CONSTR \| METHOD | DETAIL: FIELD \| CONSTR \| METHOD |  |


---


## weka.core Class Debug.Random

```
java.lang.Object
  java.util.Random
      weka.core.Debug.Random
```

**All Implemented Interfaces:**: java.io.Serializable, RevisionHandler

**Enclosing class:**: Debug

---

``` public static class Debug.Random extends java.util.Random implements java.io.Serializable, RevisionHandler ```

This extended Random class enables one to print the generated random
numbers etc., before they are returned. It can either use stdout (default)
for outputting the logging information or a Log object (level is then
INFO).

**Version:**
:   $Revision: 7519 $

**Author:**
:   FracPete (fracpete at waikato dot ac dot nz)

**See Also:**: Serialized Form

---

**Constructor Summary**

`Debug.Random()`
  
          Creates a new random number generator.

`Debug.Random(boolean debug)`
  
          Creates a new random number generator.

`Debug.Random(long seed)`
  
          Creates a new random number generator using a single long seed.

`Debug.Random(long seed,
boolean debug)`
  
          Creates a new random number generator using a single long seed.


| **Method Summary** | |
| --- | --- |
| `boolean` | `getDebug()`             returns whether to print the generated random values or not |
| `long` | `getID()`             returns the unique ID of this number generator |
| `Debug.Log` | `getLog()`             the currently used log, if null then stdout is used for outputting the debugging information |
| `java.lang.String` | `getRevision()`             Returns the revision string. |
| `boolean` | `nextBoolean()`             Returns the next pseudorandom, uniformly distributed boolean value from this random number generator's sequence. |
| `void` | `nextBytes(byte[] bytes)`             Generates random bytes and places them into a user-supplied byte array. |
| `double` | `nextDouble()`             Returns the next pseudorandom, uniformly distributed double value between 0.0 and 1.0 from this random number generator's sequence. |
| `float` | `nextFloat()`             Returns the next pseudorandom, uniformly distributed float value between 0.0 and 1.0 from this random number generator's sequence. |
| `double` | `nextGaussian()`             Returns the next pseudorandom, Gaussian ("normally") distributed double value with mean 0.0 and standard deviation 1.0 from this random number generator's sequence. |
| `int` | `nextInt()`             Returns the next pseudorandom, uniformly distributed int value from this random number generator's sequence. |
| `int` | `nextInt(int n)`             Returns a pseudorandom, uniformly distributed int value between 0 (inclusive) and the specified value (exclusive), drawn from this random number generator's sequence. |
| `long` | `nextLong()`             Returns the next pseudorandom, uniformly distributed long value from this random number generator's sequence. |
| `void` | `printStackTrace()`             prints the current stacktrace |
| `void` | `setDebug(boolean value)`             sets whether to print the generated random values or not |
| `void` | `setLog(Debug.Log value)`             the log to use, if it is null then stdout is used |
| `void` | `setSeed(long seed)`             Sets the seed of this random number generator using a single long seed. |
| `java.lang.String` | `toString()`             returns a string representation of this number generator |

**Methods inherited from class java.lang.Object**

`equals, getClass, hashCode, notify, notifyAll, wait, wait, wait`

**Constructor Detail**

### Debug.Random

```
public Debug.Random()
```

:   Creates a new random number generator. With no debugging.

---


### Debug.Random

```
public Debug.Random(long seed)
```

:   Creates a new random number generator using a single long seed.
    With no debugging

    **Parameters:**: `seed` - the seed value

---


### Debug.Random

```
public Debug.Random(boolean debug)
```

:   Creates a new random number generator. With optional debugging.

    **Parameters:**: `debug` - if true, debugging output is enabled

---


### Debug.Random

```
public Debug.Random(long seed,
                    boolean debug)
```

:   Creates a new random number generator using a single long seed.
    With optional debugging

    **Parameters:**: `seed` - the seed value: `debug` - if true, debugging output is enabled


**Method Detail**

### setDebug

```
public void setDebug(boolean value)
```

:   sets whether to print the generated random values or not

    :   **Parameters:**: `value` - if true debugging output is enabled

---


### getDebug

```
public boolean getDebug()
```

:   returns whether to print the generated random values or not

    :   **Returns:**: true if debugging output is enabled

---


### setLog

```
public void setLog(Debug.Log value)
```

:   the log to use, if it is null then stdout is used

    :   **Parameters:**: `value` - the log to use

---


### getLog

```
public Debug.Log getLog()
```

:   the currently used log, if null then stdout is used for outputting
    the debugging information

    :   **Returns:**: the log, can be null

---


### getID

```
public long getID()
```

:   returns the unique ID of this number generator

    :   **Returns:**: the unique ID of this number generator

---


### printStackTrace

```
public void printStackTrace()
```

:   prints the current stacktrace

---


### nextBoolean

```
public boolean nextBoolean()
```

:   Returns the next pseudorandom, uniformly distributed boolean value from
    this random number generator's sequence.

    :   **Overrides:**: `nextBoolean` in class `java.util.Random`
    :   **Returns:**: random boolean

---


### nextBytes

```
public void nextBytes(byte[] bytes)
```

:   Generates random bytes and places them into a user-supplied byte array.

    :   **Overrides:**: `nextBytes` in class `java.util.Random`
    :   **Parameters:**: `bytes` - array to fill with random bytes

---


### nextDouble

```
public double nextDouble()
```

:   Returns the next pseudorandom, uniformly distributed double value between
    0.0 and 1.0 from this random number generator's sequence.

    :   **Overrides:**: `nextDouble` in class `java.util.Random`
    :   **Returns:**: random double

---


### nextFloat

```
public float nextFloat()
```

:   Returns the next pseudorandom, uniformly distributed float value between
    0.0 and 1.0 from this random number generator's sequence.

    :   **Overrides:**: `nextFloat` in class `java.util.Random`
    :   **Returns:**: random float

---


### nextGaussian

```
public double nextGaussian()
```

:   Returns the next pseudorandom, Gaussian ("normally") distributed double
    value with mean 0.0 and standard deviation 1.0 from this random number
    generator's sequence.

    :   **Overrides:**: `nextGaussian` in class `java.util.Random`
    :   **Returns:**: random double, gaussian distributed

---


### nextInt

```
public int nextInt()
```

:   Returns the next pseudorandom, uniformly distributed int value from this
    random number generator's sequence.

    :   **Overrides:**: `nextInt` in class `java.util.Random`
    :   **Returns:**: random int

---


### nextInt

```
public int nextInt(int n)
```

:   Returns a pseudorandom, uniformly distributed int value between 0
    (inclusive) and the specified value (exclusive), drawn from this random
    number generator's sequence.

    :   **Overrides:**: `nextInt` in class `java.util.Random`
    :   **Parameters:**: `n` - the upper limit (exclusive) **Returns:**: random int

---


### nextLong

```
public long nextLong()
```

:   Returns the next pseudorandom, uniformly distributed long value from this
    random number generator's sequence.

    :   **Overrides:**: `nextLong` in class `java.util.Random`
    :   **Returns:**: random long

---


### setSeed

```
public void setSeed(long seed)
```

:   Sets the seed of this random number generator using a single long seed.

    :   **Overrides:**: `setSeed` in class `java.util.Random`
    :   **Parameters:**: `seed` - the seed value

---


### toString

```
public java.lang.String toString()
```

:   returns a string representation of this number generator

    :   **Overrides:**: `toString` in class `java.lang.Object`
    :   **Returns:**: a string representation

---


### getRevision

```
public java.lang.String getRevision()
```

:   Returns the revision string.

    :   **Specified by:**: `getRevision` in interface `RevisionHandler`
    :   **Returns:**: the revision


---


|  |  |  |
| --- | --- | --- |
| **Overview** \| **Package** \| **Class** \| **Tree** \| **Deprecated** \| **Index** \| **Help** \| **Weka's home** | |  |
| **PREV CLASS**   **NEXT CLASS** | **FRAMES**    **NO FRAMES**     **All Classes** |  |
| SUMMARY: NESTED \| FIELD \| CONSTR \| METHOD | DETAIL: FIELD \| CONSTR \| METHOD |  |


---
